# Effect of oesophagectomy on lipid profiles in patients with oesophageal cancer combined with hyperlipidaemia: a retrospective study

**DOI:** 10.1186/s12944-024-02091-3

**Published:** 2024-04-15

**Authors:** Jingrong Yang, Yaxin Li, Jialei Huang, Jiabin Lai, Xiangrui Chen, Wenxuan Xia, Yu Wang

**Affiliations:** 1 Department of Cardiothoracic Surgery, Fuzong Clinical Medical College of Fujian Medical University, The 900th Hospital of Joint Logistic Support Forc, PLA, Fuzhou, Fujian 350025 China; 2 The School of Basic Medical Sciences, Fujian Medical University, Fuzhou, 350122 Fujian China; 3 Department of General Surgery, Fuzong Clinical Medical College of Fujian Medical University & Dongfang Hospital of Xiamen University & The 900th Hospital of Joint Logistics Support Force, No.156 North West Second Ring Road, Fuzhou, Fujian 350025 P.R. China

**Keywords:** Oesophagectomy, Oesophageal cancer, Hyperlipidaemia, Lipids, Metabolism

## Abstract

**Background:**

Surgery is widely regarded as a pivotal therapeutic approach for treating oesophageal cancer, and clinical observations have revealed that many oesophageal cancer patients also present with concomitant hyperlipidaemia. It is surprising that few studies have been performed to determine how blood lipid levels are affected by oesophageal cancer resection. This research was designed to assess the influence of oesophageal cancer resection on lipid profiles among individuals diagnosed with both oesophageal cancer and hyperlipidaemia.

**Methods:**

A retrospective analysis was carried out on 110 patients with hyperlipidaemia and oesophageal cancer who had undergone oesophagectomy at the 900th Hospital of the Joint Logistics Support Force of the Chinese People's Liberation Army. Preoperative and postoperative serological data were collected at seven-, thirty-, sixty-day-, and one-year-long intervals. Changes in lipid levels were compared, the remission of various types of hyperlipidaemia was statistically assessed, and Pearson correlation was used to analyse the association between lipid changes and preoperative body weight. The research sought to assess the reduction in body weight and the proportion of body weight lost one year following surgery.

**Results:**

Noteworthy decreases were observed in total cholesterol (TC), triglyceride (TG), and low-density lipoprotein (LDL) levels, with TC decreasing from 6.20 mmol/L to 5.20 mmol/L, TG decreasing from 1.40 mmol/L to 1.20 mmol/L, and LDL decreasing from 4.50 mmol/L to 3.30 mmol/L. Conversely, there was a notable increase in high-density lipoprotein (HDL) levels, which increased from 1.20 mmol/L to 1.40 mmol/L (*P* < 0.05) compared to the preoperative levels. Notably, the remission rates for mixed hyperlipidaemia (60.9%) and high cholesterol (60.0%) were considerably greater than those for high triglycerides (16.2%). Alterations in TC at one year postoperatively correlated with preoperative weight and weight loss (*r* = 0.315, -0.216); changes in TG correlated with preoperative weight, percentage of total weight loss (TWL%), and weight reduction (*r* = -0.295, -0.246, 0.320); and changes in LDL correlated with preoperative weight, TWL%, and weight loss (*r* = 0.251, 0.186, and -0.207). Changes in non-high-density lipoprotein(non-HDL) were linked to preoperative weight (*r* = 0.300), and changes in TG/HDL were correlated with preoperative weight and TWL% (*r* = -0.424, -0.251).

**Conclusions:**

Oesophagectomy significantly improved lipid profiles in oesophageal cancer patients, potentially leading to a reduction in overall cardiovascular risk.

## Introduction

Oesophageal cancer, a malignancy known for its high mortality rate and poor prognosis [1], ranks as the seventh most prevalent cancer worldwide and is the sixth leading cause of cancer-related deaths [2]. Hyperlipidaemia, characterized by elevated levels of LDL or TC,poses a substantial danger element for the development of atherosclerotic cardiovascular conditions [3]. The American Lipid Association has promoted LDL and non-HDL as the main targets of long-term therapies meant to lower the risk of atherosclerotic cardiovascular conditions [4]. Parameters such as total cholesterol, the ratio of total cholesterol to HDL, the ratio of triglycerides to HDL, and the ratio of LDL to HDL have demonstrated their reliability in predicting the onset of atherosclerosis and chronic kidney disease [5]. Notably, many oesophageal cancer patients exhibit concurrent hyperlipidaemia.

Surgery has emerged as the most efficacious approach for the treatment of oesophageal cancer [6]. Oesophagectomy for this condition typically involves the liberation of the oesophagus, excision of the oesophageal tumour, elevation of the free stomach into the abdominal cavity, creation of a tubular stomach, and the establishment of an end-to-side anastomosis connecting the proximal oesophagus to the tubular stomach at the neck. This procedure shares certain similarities with bariatric surgery. According to data from 2019, the most commonly conducted bariatric surgeries, included Roux-en-Y (38.2%), sleeve gastrectomy (46%), flexible gastric banding (7.6%), and biliopancreatic diversion (5.0%) [7]. Both oesophagectomy and bariatric surgery involve partial stomach removal or a reduction in stomach volume. Studies have demonstrated that bariatric surgery can effectively address obesity-related complications, including hyperlipidaemia [8]. Research by Paulina et al. revealed that patients with hyperlipidaemia were able to discontinue or reduce medication usage following sleeve gastrectomy and adjustable gastric banding [9, 10]. Furthermore, a meta-analysis conducted by Al Khalifa et al.reported findings showed that 54% of patients achieved full resolution of hyperlipidaemia, while 83.5% experienced enhancements subsequent to sleeve gastrectomy [11]. In summary, the impacts of weight loss surgery on hyperlipidaemia have been investigated in numerous studies. However, the effects of oesophagectomy on patients with hyperlipidaemia have often been overlooked or have failed to receive adequate attention. To the best of our knowledge, the effect of oesophagectomy on hyperlipidaemia has not been documented in any literature to date.

Through systematic observation and extended monitoring of metabolic indices (TC, TG, LDL, HDL, non-HDL, TC/HDL, TG/HDL, and LDL/HDL) in a sample of hyperlipidaemic patients, this study aimed to investigate the effects of oesophageal cancer resection on hyperlipidaemic patients. Additionally, we investigated the correlations between lipid changes and preoperative body weight, postoperative body weight, and the proportion of body weight lost following surgery after one year. The aim of this study was to clarify the effects of oesophageal cancer resection on patients’ metabolic function, thereby helping to fill the current gap in the literature and provide clinicians with better postoperative management strategies to improve patients' quality of life and long-term survival.

## Methods

### Subjects

The clinical data of 110 patients with hyperlipidaemia and oesophageal cancer who underwent esophagectomies at the Joint Logistics Support Force's 900TH Hospital between January 2018 and July 2022 were compiled through a retrospective review. The patients were screened based on the following criteria for inclusion:had a diagnosis of oesophageal cancer based on pathology, had preoperative combined hyperlipidaemia,and underwent oesophageal cancer resection involving the creation of a tube stomach. The exclusion criteria included concurrent severe dysfunction of other vital organs, severe postoperative complications,c incomplete clinical data, and loss to follow-up. Notably, prior to the surgical procedures, all patients and their family members provided informed consent for the operations.

### Operation method

All patients were subjected to a preoperative multidisciplinary discussion, were indicated for surgery, and had no contraindications to surgery. Minimally invasive or open surgical approaches were used according to tumour characteristics (tumour adjacency to surrounding tissues, lymph node size and fusion, etc.) and personal history (e.g., history of previous thoracic or abdominal surgery). Patients in the minimally invasive group underwent thoracolaparoscopy, and patients in the open group underwent conventional open surgery. Surgical manoeuvres were performed as described in previous literature [12, 13]. The thoracic part of the operation included freeing the thoracic oesophagus and clearing the paraoesophageal lymph nodes, bilateral paraglottic lymph nodes, subglottic lymph nodes, paratracheal lymph nodes, lower pulmonary ligament lymph nodes, and paradiaphragmatic lymph nodes according to the American Joint Committee on Cancer(AJCC) guidelines. The abdominal operation portion included freeing the stomach, clearing the abdominal lymph nodes according to the AJCC guidelines, and creating a tube stomach (resection of a portion of the lesser curvature of the stomach through a linear cutting obturator, with a width of the tube stomach of approximately 3–6 cm). The oesophagus was severed after freeing the cervical oesophagus through a cervical incision, and the proximal oesophagus was anastomosed with the tube stomach at the cervical level on the left side of the neck.

### Data collection

Clinical data, including preoperative, 1-week postoperative,and 1-,3-,6-, and 12-month postoperative weight measurements, as well as blood biochemical results, were extracted from medical records, supplemented by information obtained through telephone interviews and microfilm follow-ups. A variety of factors, such as alanine aminotransferase (ALT), aspartate aminotransferase (AST), glutamyl transpeptidase (GGT), total cholesterol (TC), triglyceride (TG), high-density lipoprotein (HDL), low-density lipoprotein (LDL), glucose (GLU), uric acid (UA), total protein (TP), and albumin (ALB), were evaluated biochemically. The follow-up process continued until August 2022.

### Definition of hyperlipidaemia, non-HDL,and TWL%

The criteria for defining and classifying hyperlipidaemia were in accordance with the Chinese Guidelines for Prevention and Control of Dyslipidaemia in Adults (2016 Revision) [14]. According to the lipid stratification criteria, both borderline elevation and elevation were considered abnormal. Hyperlipidaemia was identified if any of the following conditions were met: (1) TC ≥ 5.2 mmol/L;(2) TG ≥ 1.7 mmol/L; (3) LDL-C ≥ 3.4 mmol/L;or (4) HDL-C ≤ 1.0 mmol/L.

Hyperlipidaemia was further classified into hypercholesterolemia, hypertriglyceridaemia, and mixed hyperlipidaemia based on clinical dyslipidaemia classification. At the one-year postoperative examination, remission of hyperlipidaemia was defined as the absence of a rise in TC, TG, or LDL, together with a reduction in at least one of these parameters.

Non-HDL represents the cumulative cholesterol content within lipoproteins other than HDL and is computed using the non-HDL-C = TC—HDL-C formula.

The TWL% was computed by dividing the disparity between the weight reported on the postoperative follow-up day and the original preoperative weight by the preoperative weight.

### Statistical analysis

The statistical software SPSS (version 20.0) was used for all analyses. Normally distributed continuous variables are expressed as the mean ± standard deviation (x ± s), while variables that do not meet these requirements are expressed as the median (quartiles) [M(P25, P75)], the fundamental clinical traits of the research population are expressed as the number of patients and percentages. The Wilcoxon paired-sign rank test was used to compare differences between the two groups in terms of the matched-pair design before surgery and one year after surgery. The Friedman test was used for repeated measures of non-normally distributed measures. The rate of remission for the various forms of hyperlipidaemia was determined using the Pearson Chi-square test. Correlations between changes in lipid levels and preoperative weight, weight loss at 1 year postoperatively, and TWL% were analysed, and the coefficient of variation was assessed by Pearson correlation if normality was satisfied, and by Spearman correlation if normality was not satisfied. *P* < 0.05 was considered indicative of statistical significance for the differences.

## Results

### Basic data about oesophageal cancer patients who also have hyperlipidaemia

Metabolic indices were available for 110 patients (46 females, 64 males) prior to oesophagectomy for esophageal cancer. Table 1 displays the characteristics of the patients. The patients ranged in age from 34 to 81 years, with an average age of 59.06 ± 8.06 years. The preoperative weight was 60.03 ± 10.60 kg, and body mass index was 22.18 ± 3.31 kg/m^2^. Nineteen patients had a history of alcohol consumption, and twenty-three had a history of smoking. Eight individuals had type 2 diabetes mellitus, twenty-six had hypertension, four had hyperuricaemia, and eight had fatty liver disease in combination. Nine of these patients underwent open heart surgery and 101 underwent minimally invasive surgery. The operation time was 195.9 ± 17.6 min, and the volume of intraoperative blood loss was 189.8 ± 46.7 mL. The total number of patients with of postoperative complications was 28.

**Table 1:** Baseline characteristics of the patients

Characteristics	Overall
Age (years)	59.06 ± 8.06
Sex (Male)	64 (58.2%)
Weight (kg)	60.03 ± 10.60
BMI (kg/m^2^)	22.18 ± 3.31
T stage	
T0	5 (4.5%)
T1	19 (17.3%)
T2	37 (33.6%)
T3	47 (42.7%)
T4	2 (1.8%)
N stage	
N0	49 (44.5%)
N1	32 (29.1%)
N2	20 (18.2%)
N3	9 (8.2%)
Clinical stage	
Stage I	20 (18.2%)
Stage II	37 (33.6%)
Stage III	42 (38.2%)
Stage IV	11 (10.0%)
Smoking	23 (20.9%)
Drinking	19 (17.3%)
Hypertension	26 (23.6%)
Diabetes	8 (7.3%)
hyperuricemia	4 (3.6%)
Fatty liver	8 (7.3%)
Type of surgery	
Minimally invasive surgery	101 (91.8%)
Open surgical	9 (8.2%)
Operation time(min)	195.9 ± 17.6
Blood loss(mL)	189.8 ± 46.7
Resection type	18.7 ± 8.3
R0	105(95.5%)
R1	5(4.5%)
R2	0
Harvested lymph node	14.78 ± 5.12
ICU stay (d)	3.56 ± 1.98
Hospital stay (d)	10.3 ± 5.7
Complication	
Recurrent laryngeal nerve injury	6 (5.5%)
Anastomotic leakage	2 (1.8%)
Anastomotic stenosis	4 (3.6%)
Postoperative hemorrhage	2 (1.8%)
Chylothorax	1 (0.9%)
Wound infection	2 (1.8%)
Respiratory complication	2 (1.8%)
Cardial complications	1 (0.9%)
Gastric emptying dysfunction	5 (4.5%)
Diaphragmatic hernia	3 (2.7%)
Total	28 (25.5%)

The data are shown as a number (%) or as the mean ± SD

*BMI* body mass index, *kg* kilogram

### Changes in serological indicators in patients 1 year after oesophagectomy for oesophageal cancer

The results of 13 serologic indices before and 1 year after surgery are shown in Table 2. One year after surgery, the serum TC, TG, LDL, ApoB, TP, ALB, and UA levels were significantly decreased (*P* < 0.01), the AST, GLU, ALT, and HDL levels were considerably elevated (*P* < 0.01), apolipoprotein A1 (ApoA1) levels were decreased, and GGT levels were increased. However, these changes were not statistically significant.

**Table 2:** Changes in the serum indices from before to one year after operation

Parameters	Pre-operation	1 year after operation
TC (mmlo/l)	6.20(5.60,6.73)	5.20 (4.78, 5.63)*
TG (mmol/l)	1.40(1.00,2.40)	1.20(0.90,1.60)*
HDL(mmol/l)	1.20(0.98,1.40)	1.40(1.20,1.60)*
LDL (mmol/l)	4.50(3.90,4.90)	3.30(2.88,3.80)*
ApoA1(mmol/l)	1.10(1.00,1.30)	1.20(1.00,1.40)
ApoB(mmol/l)	1.30(1.20,1.50)	1.00(0.90,1.23)*
GLU(mmol/l)	5.20(4.90,5.90)	6.40(5.00,8.13)*
TP(g/l)	72.70(69.58,74.95)	69.80(65.20,73.73)*
ALB(g/l)	44.05(40.75,46.08)	42.30(38.68,44.63)*
UA(μmol/l)	349.65(299.1,410.35)	323.45(256.35,368.90)*
ALT(U/l)	15.55(12.08,23.15)	26.25(13,38,46.33)*
AST(U/l)	17.70(14.50,22.05)	22.90(15.58,34.53)*
GGT(U/l)	27.10(19.00,42.10)	33.30(16.68,55.13)

The data are presented as the median (IQR); *IQR* interquartile range

^*^P < 0.01

### Alterations in lipid levels among patients following oesophagectomy for oesophageal cancer

**Fig. 1:**
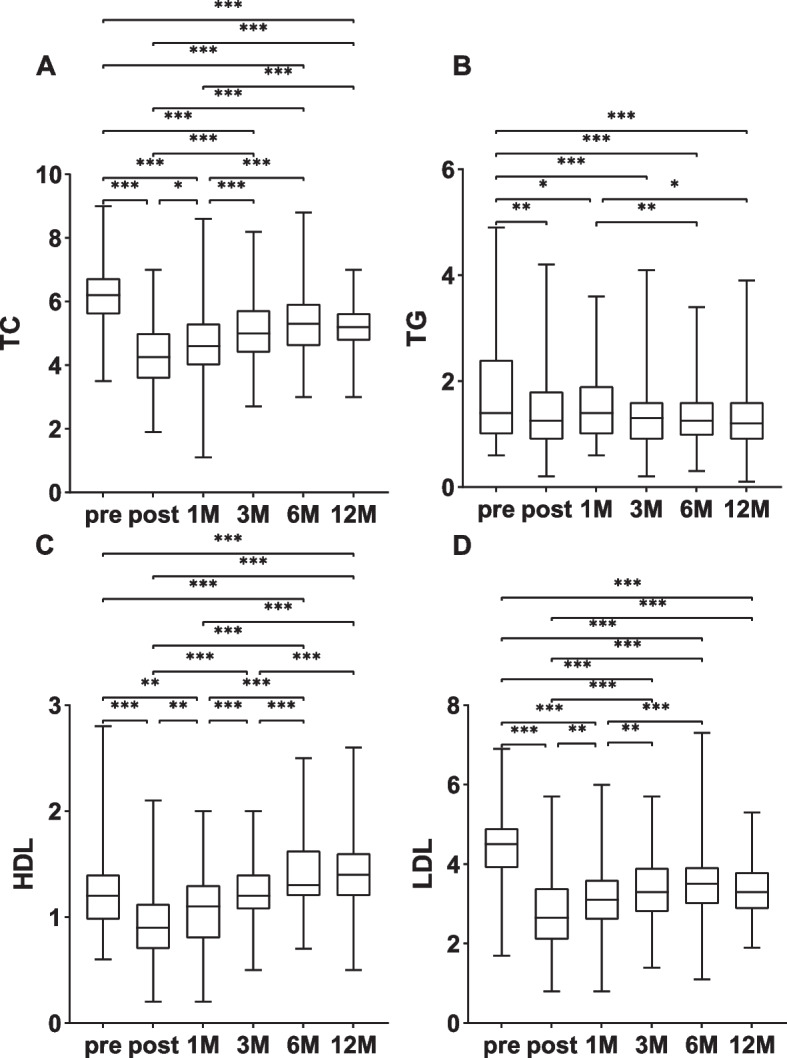
Follow-up of TC, TG, HDL, and LDL in hyperlipidaemic patients. **A **TG; **B **TC; **C **HDL; **D **LDL. Comparison of two groups, ***P < 0.001; **P < 0.01; *P < 0.05. pre, preoperative; post, post-operative; 1M, one month following the procedure; 3M, three months following the procedure; 6M, six months following the procedure; 1Y, one year following the procedure

One week after surgery, the TC levels considerably decreased from their preoperative values (6.20 mmol/L vs. 4.25 mmol/L, *P* < 0.001), then slowly increased over time and rose to a maximum at 6 months postoperatively but were still lower than the preoperative levels (6.20 mmol/L vs. 5.30 mmol/L, *P* < 0.001). Between one year and six months following surgery, there was no discernible change in total cholesterol levels (Fig. 1A). TG levels decreased significantly at 1 week postsurgery (1.40 mmol/L vs. 1.25 mmol/L, *P* < 0.01), but there was a short-term increase at one month postsurgery, followed by a gradual decrease, after which TG levels were significantly lower than the preoperative level at one year postsurgery (1.40 mmol/L vs. 1.20 mmol/L, *P* < 0.001, Fig. 1B). Serum HDL levels decreased significantly from 1.20 mmol/L to 0.90 mmol/L at 1 week postsurgery (*P* < 0.001), increased gradually over time and were significantly greater than the preoperative levels at 1 year postsurgery (*P* < 0.001, Fig. 1C). LDL levels decreased significantly at 1 week postoperatively (4.50 mmol/L vs. 2.65 mmol/L, *P* < 0.001) and then gradually increased to the highest level at 6 months postoperatively, which was significantly different from the preoperative level (4.50 mmol/L vs. 3.50 mmol/L, *P* < 0.001) subsequently, the difference observed at one year and six months following surgery was not statistically significant (Fig. 1D).

**Fig. 2:**
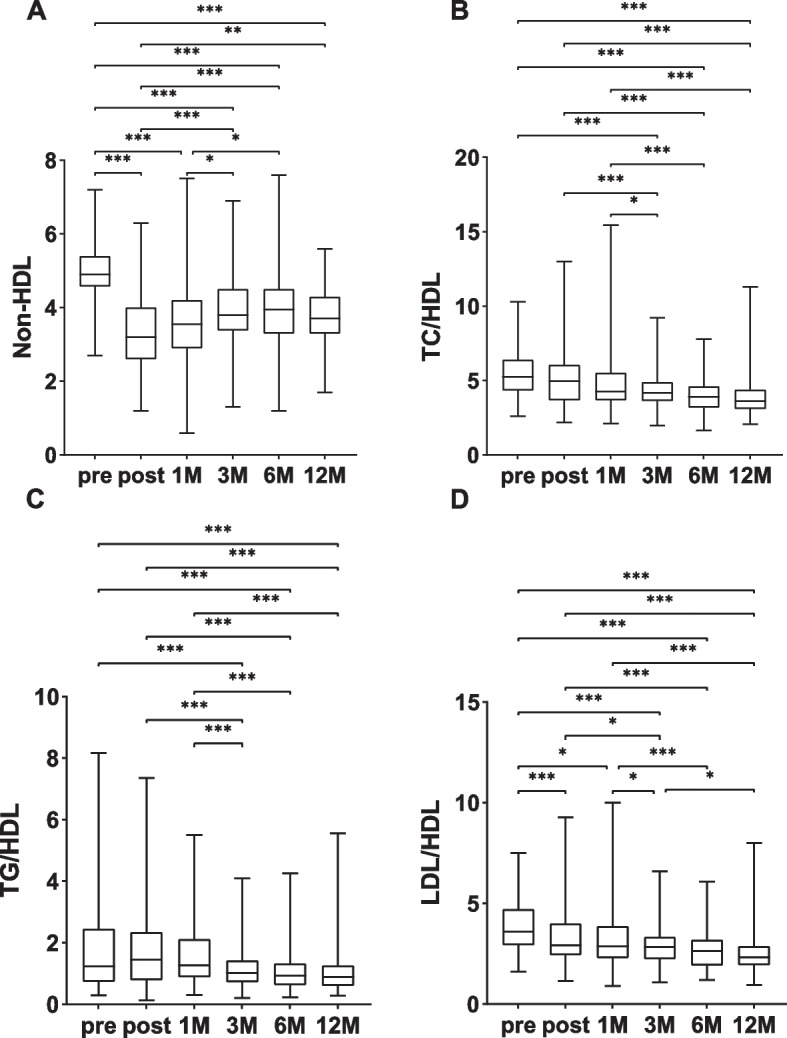
Follow-up of non-HDL, TC/HDL, TG/HDL, and LDL/HDL in hyperlipidaemic patients. **A** non-HDL; **B** TC/HDL; **C** TG/HDL; **D** LDL/HDL. Comparison of two groups, ***P < 0.001; **P < 0.01; *P < 0.05. Pre, preoperativ; post, postoperative; 1M, one month following the procedure; 3M, three months following the procedure; 6M, six months following the procedure; 1Y:one year following the procedure

The non-HDL level was 4.90 mmol/L preoperatively and decreased significantly to 3.20 mmol/L one week postoperatively (*P* < 0.001) it gradually increased at 1 month postoperatively and reached the highest level at 6 months postoperatively, which was significantly different from the preoperative level (4.90 mmol/L vs. 3.95 mmol/L, *P* < 0.001, Fig. 2A). The postoperative TC/HDL ratio continued to decrease, with no statistically significant difference between the preoperative levels one week and one month after surgery; three months after the operation, the ratio of total cholesterol to HDL cholesterol was substantially lower than the preoperative levels (5.43 vs. 4.28, *P* < 0.001) and then gradually declined over time, but there there was no noticeable difference from 3 months postoperatively (Fig. 2B). TG/HDL was elevated for a short period of time, 1 week postoperatively, and then 1 month postoperatively but was not significantly different from the preoperative comparison; moreover, TG/HDL was significantly lower than the preoperative level at 3 months postoperatively (1.74 vs. 1.16, *P* < 0.001) and was not significantly different over time (Fig. 2C). LDL/HDL showed a continuous decreasing trend and decreased to the lowest level at 1 year postoperatively, which was significantly different from the preoperative level (3.85 vs. 2.50, *P* < 0.001 Fig. 2D).

### Remission of patients with different types of hyperlipidaemia 1 year after surgery

Based on the preoperative lipid levels, 110 patients were categorized into the hypercholesterolemia group (*n* = 70), the high triglyceride group (*n* = 12), and the mixed hyperlipidaemia group (*n* = 28) according to the clinical classification of China's Guidelines for Adult Dyslipidaemia Prevention and Management (Revised Edition 2016). The remission rates varied by a significant margin among the three groups (*P* < 0.01). The remission rate in the high-cholesterol group (60.0%) was greater than that in the high-triglyceride group (16.2%), and the remission rate in the mixed-hyperlipidaemia group (60.9%) was greater than that in the group with high triglycerides (16.2%). All of these findings were extremely noteworthy (*P* < 0.05), and no extremely noteworthy variation was detected in the remission rate between the high-cholesterol group and the mixed-hyperlipidaemia group (*P* > 0.016667).

### The relationship between lipid changes and weight changes 1 year after surgery

We investigated the correlation between changes in lipid levels and preoperative weight, weight loss, and TWL% (Table 3). Changes in cholesterol 1 year after surgery were found to be positively correlated with preoperative body weight (*r* = 0.315, *P* < 0.05) and adversely connected with weight loss (*r* = 0.216, *P* < 0.05); changes in triglycerides 1 year after surgery were negatively correlated with preoperative body weight and TWL% (*r* = 0.295, 0.246, *P* < 0.05) and favourably related to weight loss (*r* = 0.320, *P* < 0.05); changes in LDL at 1 year postoperatively were favourably connected with the weight before surgery and TWL% (*r* = 0.251, 0.186, *P* < 0.05) and inversely connected with weight loss (*r* = 0.207, *P* < 0.05); changes in non-HDL were favourably connected with preoperative weight (*r* = 0.300, *P* < 0.05); and changes in TG/HDL were negatively correlated with TWL% (*r* = 0.424, 0.251,*P* < 0.05).

**Table 3:** Variations in lipid levels and preparation weight, weight loss, and TWL% one year following surgery are correlated

Lipid parameters	Pre-op weight		Weight loss		TWL%	
	*r*	*P*	*r*	*P*	*r*	*P*
TC (mmol/l)	0.315	0.001*	-0.216	0.024*	0.157	0.102
TG (mmol/l)	-0.295	0.002*	0.320	0.001*	-0.246	0.010*
HDL(mmol/l)	0.092	0.340	0.067	0.489	-0.088	0.362
LDL (mmol/l)	0.251	0.008*	-0.207	0.030*	0.186	0.052
Non-HDL(mmol/l)	0.300	0.001*	-0.231	0.015*	0.178	0.063
TC/HDL	0.037	0.703	-0.127	0.186	0.121	0.210
TG/HDL	-0.424	0.000*	0.346	0.000*	-0.251	0.008*
LDL/HDL	0.071	0.458	-0.124	0.195	0.119	0.214

*TWL%* Total weight loss percentage

^*^P < 0.05

## Discussion

This retrospective investigation included 110 patients who were diagnosed with oesophageal cancer coupled with hyperlipidaemia and who underwent oesophagectomy for the management of their oesophageal malignancy. The analysis conducted in the study highlighted a significant improvement in lipid profiles following oesophagectomy that could help reduce the risk of cardiovascular diseases. Variations in the extent of improvement were observed among different subtypes of hyperlipidaemia, with mixed hyperlipidaemia exhibiting the most pronounced remission rate. Further scrutiny revealed a discernible correlation between alterations in lipid parameters and weight reduction.

The beneficial influence of bariatric surgery on lipid homeostasis has been substantiated by a wealth of literature. Recent investigations have demonstrated that bariatric procedures substantially ameliorate lipid profiles, manifesting as a cardio-protective synergy involving reductions in TC, LDL, TG, and elevated HDL [15]. In concordance, a comprehensive review revealed that one year postbariatric surgery, patients exhibit reduced LDL and TG levels, elevated HDL levels, diminished left ventricular dimensions and mass, and heightened ejection fractions [16]. Kjellmo CA et al. reported that bariatric surgery elicited a favourable modulation of lipoprotein profiles among morbidly obese individuals, characterized by lowered LDL and apoB levels, an attenuated SAA/PON1 ratio, and augmented HDL concentrations. Nevertheless, this intervention did not affect cholesterol efflux capacity [17]. Moreover, retrospective inquiries have alluded to a diminished cardiovascular disease risk among individuals undergoing laparoscopic sleeve gastrectomy, manifesting as a substantial 12.7% increase in HDL levels and a 22.9% decrease in TG levels [18]. Correspondingly, the findings in the study are consistent with these observations. In an investigation involving 110 patients with oesophageal cancer and hyperlipidaemia who underwent oesophagectomy, a notable 16.7% increase in HDL levels was observed one year postprocedure. Conversely, TG, LDL, and non-HDL levels decreased by 14.3%, 26.7%, and 23.2%, respectively. In summary, both oesophagectomy and bariatric surgery have demonstrated lipid ameliorative effects.

To discern a rationale for the improvements in lipid profiles, it is pertinent to note that plasma gastrin levels exhibit a noteworthy reduction subsequent to laparoscopic sleeve gastrectomy. This reduction correlates with a diminished appetite and decelerated gastric emptying, subsequently fostering effective weight loss during the postoperative period [19]. These mechanisms may underpin the observed alterations in HDL and TG levels during the postoperative phase. Notably, parallels exist between the surgical manoeuvres employed in oesophageal cancer resection and sleeve gastrectomy. Several studies have elucidated the utility of the tube stomach technique in oesophagectomy for oesophageal cancer. Some scholars posit that the diameter of the tube stomach closely approximates that of the oesophagus. This physiological feature reduces the food retention time within the thoracic stomach, bolstering peristaltic activity and preventing thoracic-gastric syndrome attributed to gastric retention. Moreover, partial gastric tissue resection during these surgeries curtails gastric acid secretion, consequently preventing postoperative reflux oesophagitis. Furthermore, the tube stomach technique has been shown to effectively diminish the incidence of postoperative respiratory and circulatory complications [20]. This finding emphasizes that both oesophagectomy and sleeve gastrectomy entail partial gastric tissue removal, which results in a reduction in gastric volume. This, in turn, diminishes gastric lipase secretion as well as the release of cholecystokinin, a hormone known to stimulate the secretion of digestive enzymes such as lipases and proteases. These cumulative effects culminate in a marked reduction in triglyceride hydrolysis and attenuated absorption of free fatty acids, thereby enhancing overall blood lipid profiles [21]. However, despite the commonality of i lipid level reductions following these procedures, the magnitude of reduction may vary contingent on the location and extent of gastric tissue resection. Notably, during sleeve gastrectomy, a substantial section of the fundus—the main location of gastrin secretion—and the stomach's larger curvature are removed [22]. Gastrin has been implicated in stimulating appetite, augmenting gastric motility, promoting growth hormone secretion, and inhibiting fat utilization. The removal of the fundus of the stomach also perturbs the release of intestinal hormones, including gastrin [23]. Conversely, oesophageal carcinoma resection targets a segment of the lesser curvature of the stomach, exerting a comparatively milder influence on intestinal hormones, consequently resulting in a lesser reduction in blood lipid levels.

In the dynamic analysis of lipid fluctuations among patients with oesophageal cancer and concurrent hyperlipidaemia at the one-year postoperative mark, a significant reduction in all lipid parameters was observed within the initial week following oesophageal cancer resection. This intriguing phenomenon can be attributed, in part, to the dietary shifts enforced during the postoperative period. Notably, at the institution, patients diagnosed with oesophageal cancer typically adhere to a regimen of fasting with water intake for approximately 5–7 days following surgery. Only after confirmation of the absence of critical complications, such as anastomotic fistula, are pacients permitted to initiate oral intake. In the initial phases of dietary reintroduction, most patients primarily adhere to a liquid diet. Furthermore, Jahansouz et al. reported that acute changes in the expression and activity of peroxisome proliferator-activated receptor delta (PPARδ) and gamma (PPARγ) in subcutaneous adipose tissue are caused by bariatric surgery. These changes correlate with a reduction in lipid storage, an increase in lipolysis, and an increase in lipid oxidation [24]. Correspondingly, the study revealed a transient decrease in serum TC and LDL levels in patients following oesophageal cancer resection. This could be linked to a similar mechanism.

In addition to dietary influences and the metabolic consequences of surgery, it is pertinent to consider the role of the surgical stress response. Surgical stimuli have been associated with increased cortisol and growth hormone release through the hypothalamic-pituitary axis, which, in turn, fosters lipolysis [25]. As time progresses, the effects of these factors on lipid profiles gradually wane or diminish, resulting in a gradual increase in lipid levels beginning one month postoperatively. Notably, TC and LDL levels peak at the six-month postoperative follow-up, albeit remaining significantly lower than the preoperative levels. Conversely, HDL levels reach their zenith at the one-year postoperative milestone, significantly surpassing preoperative levels. Intriguingly, there were no significant differences in TG levels between the first week and subsequent postoperative periods.

This finding is in contrast to that of a study by Magdalena Vila et al., who examined the lipid profiles of patients after pancreaticobiliary bypass. Their findings indicated a significant decrease in TC levels postsurgery, with a nadir being reached at one year postsurgery, followed by a gradual increase. Conversely, TG levels exhibited an increase from one month postoperatively to six months postoperatively, followed by a decrease, with a substantial decrease observed at one year postoperatively. HDL decreased at three months postsurgery, followed by a significant increase at the six months and one year postsurgery. LDL levels continued to decrease postoperatively, with the most pronounced reduction occurring at three months postoperatively. Discrepancies with the study's findings could be attributed to variances in the timing of follow-up assessments, with Magdalena Vila et al. commencing their postoperative evaluation at three months as opposed to the assessment at one week postoperatively in this study.

Numerous scholars have investigated the correlation between postoperative lipid alterations and weight loss after bariatric surgery. Dattilo AM et al. have posited that weight loss is closely linked with noteworthy reductions in TC (*r* = 0.32), LDL (*r* = 0.29), very-low-density lipoprotein (*r* = 0.38), and TG (*r* = 0.32) [26]. Researchers Aucott L et al. established a linear association, showing that cholesterol decreases by 1.3% and triglycerides decreased by 1.6% for each kilogram of weight reduction [27]. Regrettably, pertinent studies exploring the interface between lipid alterations and weight loss following oesophageal cancer resection are lacking. In this investigation, an attempt was made to bridge this knowledge gap by analysing the correlation of lipid fluctuations with changes in weight among patients who underwent oesophagectomy for oesophageal cancer. This findings revealed that alterations in TC, LDL, and non-HDL levels at one-year postoperative juncture were positively correlated with preoperative body weight and a negatively correlated with one-year postoperative weight loss. Conversely, TG fluctuations exhibited an inverse relationship with preoperative body weight and TWL% and a positive correlation with one-year postoperative weight loss. Similarly, regression analysis by L. N. Zhang et al. revealed a positive correlation between body weight changes and alterations in TC, LDL, and TG, along with a negative correlation with HDL fluctuations. Their findings implied that irrespective of baseline weight, weight loss confers benefits in terms of improving blood pressure, glucose profiles, and lipid parameters among middle-aged and older adults [28]. Furthermore,according to Busetto L et al., in morbidly obese people, slight weight loss—roughly 10–20% of starting body weight—has the greatest effect on lipid profiles [29]. This finding substantiates the close association between weight loss and lipid improvements. The underlying rationale for this association may be attributed to heightened utilization of lipid reserves within adipose tissue that involves fat consumption for energy and consequently reduction of blood lipid levels. Notably, this study further revealed that changes in the TG/HDL ratio were negatively correlated with preoperative body weight and TWL% but positively correlated with one-year postoperative weight loss. A possible early indicator of insulin resistance is the TG/HDL ratio. Recent investigations have suggested that caloric restriction prompts hepatic fat depletion within one week, subsequently enhancing hepatic insulin sensitivity and clearance, irrespective of whether metabolic surgery is performed [30, 31].

Following oesophagectomy, patients may encounter unintended reductions in weight and gastrointestinal complaints linked to inadequate nourishment and impaired functional recovery, even in the absence of long-term recurrence [32]. Furthermore, malnourished patients may develop sarcopenia and osteoporosis alongside weight loss [33]. The multifactorial causes of weight loss include tumour-related symptoms (e.g., dysphagia), metabolic abnormalities, treatment-related toxicity, and muscle atrophy due to patient inactivity [34]. Studies by Scarpa et al. and Wu et al. have demonstrated the enhancement of postoperative nutritional profiles, including loss of appetite and body mass index, through minimally invasive oesophagectomy [35, 36]. Postesophagectomy enteral nutrition commonly involves the use of a jejunostomy tube. Adequate nutritional support is essential for in preventing malnutrition in oesophagectomy patients, and enteral nutrition rich in eicosapentaenoic acid (EPA) has been shown to preserve lean body mass postesophagectomy [37]. Additionally, a study reported that an enteral diet rich in ω-3 fatty acids improved oxygenation post-thoracic oesophagectomy [38]. A walking-feeding intervention for patients with newly diagnosed locally advanced oesophageal cancer during neoadjuvant chemoradiation therapy initiation and completion effectively maintained patients' functional walking ability and nutritional status [39]. Nutritional interventions primarily focus on providing adequate calories and nutrients through additional supplements or educating patients to opt for a protein- and calorie-rich diet [40, 41]. Long-acting octreotide has been utilized for decades to reduce postprandial symptoms and induce weight gain in various clinical scenarios related to reducing body weight [42]. Surgical growth inhibitor analogues have also been employed to alleviate postprandial symptoms and may have a favourable impact on nutrition, improving the recovery of weight loss following surgery [43].

There are a few noteworthy strengths in this study. First, the study focused on patients with oesophageal cancer combined with hyperlipidaemia, a subject which has reveived less attention in existing scientific research. By studying the relationship between preoperative combined hyperlipidaemia and postoperative metabolic function, preoperative management and postoperative rehabilitation can be improved. Second, the comparison of lipid changes at different postoperative time points enhances the comprehensiveness of the findings and provides valuable insights into the dynamics of lipid parameters, contributing to a deeper understanding of the long-term effects of oesophageal cancer resection on lipids. In addition, the results of this study are not only applicable to patients with oesophageal cancer, but also might additionally serve as a resource for the management of allied illnesses. This study has certain limitations. First, 110 patients from a single medical facility constituted a numerically unremarkable but eligible sample used in the study's retrospective review. It is possible that some patients did not strictly follow the requirement of fasting for 10–12 h prior to the examination, possibly leading to bias and limited the generalizability of the results to the larger group of patients with combined hyperlipidaemia undergoing oesophagectomy for oesophageal cancer. Therefore, a large sample, multicentre study is necessary to validate the results. Second, this study had a limited follow-up period, and a longer-term assessment of metabolic markers and general health would help to provide a more thorough grasp of the effects of oesophageal cancer surgery on patients with hyperlipidaemia. Third, this study focused on metabolic changes after surgery and did not delve into specific dietary and lifestyle measures that affect patients' metabolic markers after surgery. Future studies could take specific nutritional and exercise interventions into account to explore their potential impact on patients' postoperative metabolic health.

## Conclusions

In conclusion, this research endeavours were directed towards scrutinizing the impact of oesophageal cancer resection on blood lipid profiles. By systematically analysing and contrasting serological parameters in patients with oesophageal cancer and concurrent hyperlipidaemia before and after surgical intervention, researchers have gained valuable insights into this interplay. As such, researchers have posited that oesophageal cancer resection may exert a certain degree of efficacy in ameliorating hyperlipidaemia, thereby potentially reducing the risk of atherosclerotic cardiovascular disease in affected individuals. Confirmation of this effect could provide clinicians with better postoperative management strategies to enhance patients' standard of living and long-term survival. Nonetheless, it is imperative to approach these findings with a degree of circumspection, given the study's limitations, and advocate for further high-quality, long-term investigations to corroborate and extend the understanding of this complex relationship.

## Data Availability

The primary data for this study is available from the corresponding author (Yu Wang) on reasonable request.

## Abbreviations

TC: Total cholesterol
TG: Triglycerides
LDL: Low density lipoprotein
HDL: High density lipoprotein
TWL%: Total weight loss percentage
ASCVD: Atherosclerotic cardiovascular disease
AJCC: The American Joint Committee on Cancer
non-HDL: Non-high-density lipoprotein
ALT: Alanine aminotransferase
AST: Aspartate aminotransferase
GGT: Glutamyl transpeptidase
GLU: Glucose
UA: Uric acid
TP: Total protein
ALB: Albumin
ApoA1: Apolipoprotein A1
ApoB: Apolipoprotein B
